# Supplementary material for: Financial development, technological innovation and urban-rural income gap: Time series evidence from China
Source: PLoS One. 2023 Feb 10;18(2):e0279246. doi: 10.1371/journal.pone.0279246 (PMC9916642; doi:10.1371/journal.pone.0279246)
Supplement: Attachment — Submitted filename: Response to reviewers.doc [file pone.0279246.s003.doc]

Thank you for your letter and for the reviewers' and editors' comments concerning our manuscript. These comments are very helpful for revising and improving our paper. We have studied comments carefully and have made corrections which we hope meet with approval.

**Reviewer #1comments1:**

Why the authors think that FKC estimation will be a suitable theoretical framework for addressing such a research question? The objective of the study as stated by the authors is "to focus on verifying the validity of the FKC hypothesis and the dynamic relationship between technological innovation and the urban-rural income gap in the FKC hypothesis". It goes back to the first comment I have given. Authors are basically carrying out a FKC estimation study, without having any solid policy relevance.

**Response:** Thanks for your valuable comments and suggestions. We have revised and supplemented the introduction, including the necessity of this study, the policy issues to be solved by the study, and the reasons for the selection of research samples. To stimulate research. At the same time, On the basis of the estimation study of FKC hypothesis, some relevant and reliable policies are supplemented. The details are as follows:

**Introduction**

Income distribution has always been a popular issue in entire society. A reasonable income distribution system and income gap are not only the common aspiration of the people but also an important embodiment of social justice. China's urban-rural dual structure problem results from the country's policy choice in the process of reform and opening up. The country's city priority development strategy and the opening-up pilot program of coastal cities and regions have attracted an influx of talents and funds from inland areas, which has led to further disparities in income distribution between urban and rural areas and regions. Especially in recent years, with the widening gap between urban and rural residents' income growth rate, the income gap between urban and rural areas in China continues to increase, which has become an obstacle to the long-term healthy and stable development of China's economy. The data from China Statistical Bureau show that the income ratio of urban and rural residents in China has decreased from 3.30: 1 in 2007 to 2.64: 1 in 2019, and the overall trend is declining. However, compared with the world level, the income gap between urban and rural areas in China is hovering at a high level and shows a fluctuating trend. During 2002-2019, it expanded at a high level before 2009, and then fell within a narrow range, showing an inverted U-shaped trend. The Fifth Plenary Session of the 19th Central Committee of the Communist Party of China proposed the long-term goals for 2035, including 'more obvious and substantial progress in common prosperity for all people' and 'per capita GDP reaches the level of moderately developed countries, and the middle-income group has significantly expanded'. At present, the government has introduced a variety of agricultural financial policies to promote rural economic development, and gradually relaxed the household registration management system to strengthen labor mobility. However, under the current urban-rural dual structure system, the income distribution of residents in China is still very serious, and the income gap between urban and rural residents is expanding, which will inevitably affect social harmony and stability. Therefore, in realizing the path of establishing and improving the institutional mechanism of urban-rural integration development and balancing urban-rural integration development put forward in the 19th National Congress of the Communist Party of China, solving the urban-rural income gap has become the key point the sustainable economic development at this stage and in the future for a long time.

Following Kuznets [1], Greenwood and Jovanovich [2] first reveal an inverted U-shaped relationship between financial development and income inequality. Namely, with the development of finance, the income gap shows an inverted U-shaped feature of early expansion and then reduction, which is the financial Kuznets curve (FKC) hypothesis. In the early 1990s, the income gap between countries in the world was highlighted, and the mechanism of financial development on income distribution attracted wide attention in academia. At present, the research in this area has become a research frontier of financial development theory. Looking at the actual situation of our country, since the reform and opening up, the financial market has developed rapidly, the reform of the financial industry has achieved positive development, and the innovation of the industry has gradually deepened. In 2018, the scale of social financing reached 200.75 trillion yuan in the entire year, and the scale of the interbank bond market leaped to the second in the world. However, with the rapid development of financial markets, accompanied by the widening income gap between urban and rural residents, the problem of unbalanced and inadequate development still exists. Furthermore, technological innovation is considered to be the major factor affecting macroeconomic variables. Hou Zhenmei, Tian Mao, et al. [3] pointed out that the process of technological innovation is an economic growth process that takes technology as the mainline and causes social change and technological innovation is a key driving force for economic growth, and its development process will inevitably affect the change of income distribution pattern of different groups. Pan et al. [4], Wang and Wang [5] dwell on the influence of technological innovation on energy efficiency. Technological innovation can influence environmental degradation. Recently, the effect of innovation on the income gap is an important field of research [6].

The China's economy is an essential case. In 2016, China became the first middle-income economy to enter the top 25 of the global innovation index. China's scientific and technological innovation capability was enhanced and the main scientific and technological innovation indicators were steadily improved in 2018. The state intellectual property office of the data shows the proportion of research and experimental development expenditure of the entire society in GDP in 2018 was 2.15%. The total number of R&D personnel reached 4.18 million person-years, ranking first in the world; The number of international scientific papers and citations ranked second in the world; The number of patent applications and authorization of invention ranks first in the world; The contribution rate of scientific and technological progress is expected to exceed 58.5%, and the national comprehensive innovation ability ranks 17th in the world. The level of scientific and technological innovation in China has been continuously improved, and fruitful achievements have been made in many fields such as innovation input, innovation output, and innovation efficiency. These developments show technological innovations advance rapidly in China. In addition, income distribution has been a vital matter in China for a long time. The report of the 19th National Congress of the Communist Party of China pointed out that my country's 'urban and rural regional development and income distribution gap is still large', which must be solved. In this context, to incorporate technological innovation into the important factors that affect the income gap between urban and rural areas, and to explore ways and channels to ease and eliminate the excessive income gap in residential areas in China, which is of great significance to reduce income inequality and realize social equity at this stage.

Considering all the above explanations, the present study creates the following questions: First, is the FKC hypothesis valid for China? Second, how technological innovation influences income gap in China's economy? Third, is there a causal linkage between technological innovation and urban-rural income gap? In addition, there are no studies on the causal linkage between them. In this regard, this is the first study on China's economy. To answer these questions above, we attempt to make an empirical study on the link between technological innovation and income gap in China from 1985 to 2019 under the context of FKC hypothesis. We intensify on the long-term and causal relations between the variables. In the study of financial development and the income gap between urban and rural residents, this paper adds the index of technological innovation. By citing these three variables, hope to be more comprehensive on the causes of China 's urban-rural income gap and how to solve this problem are discussed, and thus draw more accurate results and set forward more constructive suggestions. The widening income gap between urban and rural areas in China has attracted wide attention from the government and academia, but from the domestic existing literature, it focuses on the role of law decision-making factors. There is no analysis on the impact of technological innovation on the income gap under the background of FKC hypothesis. In addition, the utility research on the long- and short-term causality test between technological innovation and income gap is indeed more inadequate. Thus, the study intends to focus on verifying the validity of the FKC hypothesis and the dynamic relationship between technological innovation and the urban-rural income gap in the FKC hypothesis. It provides the theoretical basis and policy suggestions for further deepening rural financial reform and technological innovation and giving full play to the role of financial services and technological innovation in the allocation of rural resources. This newly explains the causes of the urban-rural income gap, which is not only related to the overall promotion of the coordinated development of urban and rural areas but also the determination of the focus and specific direction of future financial and technological innovation development in various regions. Therefore, it is an important subject worthy of in-depth study and testing.

Thanks!

**Reviewer #1comments2:**

The research gaps are vague. The findings of FKC estimation studies are already inconsistent, and the present study conveniently adds to that inconsistency. Moreover, merely adding a variable in the framework cannot be considered as a valid research gap.

**Response:** Thanks for your valuable comments and suggestions. According to the research framework, core variables and research significance of this study, my views are as follows:

1）In the study of financial development and urban-rural income gap, there are three dominant views: i) Inverted U-shaped action view. Greenwood and Jovanovich first reveal an inverted U-shaped relationship between financial development and income inequality. Namely, with the development of finance, the income gap shows an inverted U-shaped feature of early expansion and then reduction, which is the financial Kuznets curve (FKC) hypothesis. ii) Expand the role of view. Based on the extension of Greenwood and Jovanovich model, Calor and Zeira, Banerjee and Newman put forward the view that capital market imperfections will widen the income gap. iii) Inhibition view. Chakraborty and Ray proposed that the bank dominated financial system is conducive to narrowing the income gap.

2）Innovation is the long-term driving force of economic growth and has a profound impact on economic development. Technological innovation becomes the core strategy of regional economic transformation and development. Exploring the impact of technological innovation on the urban-rural income gap is of some significance. Technological innovation is considered being the major factor affecting macroeconomic variables. However, the existing studies do not consider technological innovation as a factor determining income inequality in term of the FKC. Thus, due to deficiencies in the literature, the present study aims at examining the relationship between technological innovation and urban-rural income gap in the presence of FKC hypothesis for China economy. Therefore, only the core variable of technological innovation is added as the key analysis object of the study.

Thanks!

**Reviewer #1comments3:**

The reduced form model is susceptible to omitted variable bias and multicollinearity issues. These issues are not addressed.

**Response:** Thanks for your valuable comments and suggestions. According to the model selection, omitted variable bias and multicollinearity related problems in the manuscript, my views are as follows:

The empirical strategy of this study mainly consists of four different stages. We first apply the ADF and PP unit root tests presented by Dickey and Fuller, Phillips and Perron and Elliott et al. for stationary analysis.

Second, the cointegration methods（ARDL）proposed by Pesaran et al. are used to determine the cointegration between the series. From the research of Wang Jing, we can see that the ARDL test is superior to other classical cointegration tests the approach does not require the variable data to be a single integer sequence of the same order. After determining the optimal lag order, ARDL can analyze the long-term relationship of variables regardless of whether the variables are I(0), I(1) or mixed sequences.

Third, the long-run coefficients are estimated through the DOLS, FMOLS, and CCR estimation methods developed by Stock and Watson, Phillips and Hansen, and Park, respectively. According to Zhang Yantao's research, the DOLS technique is an asymptotically effective estimator that eliminates autocorrelation, and simultaneity problems in the cointegration equation. The FMOLS has the advantage of correcting autoregression and endogeneity problems. It is well known that omitted variables are one of the causes of endogeneity problems, so the model in this study will not be affected by omitted variable bias.

In the last stage, the causal relations between the series are analyzed by the VECM Granger causality method presented by Engle and Granger. Engle and Granger（1987）add the error correction term (ECT) in the classical VAR model as an additional variable. It has the advantage of being able to analyze the long-term and short-term causality between variables.

According to the relevant books on econometrics and website data, the application of the VECM model does not need to consider the problem of multicollinearity. The reasons are as follows：

i) The problem of multicollinearity itself affects the significance of a single factor, not the significance of the whole model. The purpose of the VECM model is not to explore the influence of individual factors on endogenous latent variables, but to analyze their comprehensive influence on endogenous latent variables as a whole, which is the key to the problem.

ii) The original intention of the VAR model is to construct the model by taking each endogenous variable as the lag value of all endogenous variables in the system, regardless of the economic theory itself.

Thanks once more!

**Reviewer #1comments4:**

The discussion is superficial, just like the previous version.

**Response:** Thanks for your valuable comments and suggestions. We have added economic intuitions and policy discussion behind the results. The details are as follows:

**VECM Granger causality test**

On the basis of the above test results, in the case of the optimal lag order of order 1, this section will continue to test the long-term and short-term causality of △lnGI、△lnTI and △lnFIR based on VECM. This method verifies the causality between variables in the composite system, which can avoid the disadvantage that the traditional Granger causality test can not be applied to the cointegration test. The causality results presented in Table7 suggest that financial development and income inequality cause each other. This conclusion is the same as that of Zhang Yingli and Yang Zhengyong [41]. He uses the VECM model to dynamically analyze the relationship between financial development, urbanization, and the urban-rural income gap. The empirical results show that financial development is the uni-directional Granger cause of the urban-rural income gap. The causal relationship results also show that there is a bidirectional causal linkage between technological innovation and urban-rural income gap at the significance levels of 1% and 5%. Ma Lei explored the impact of human capital structure and total factor productivity on urban-rural income gap from the perspective of innovation-driven development [27]. However, there is no research on the causal relationship between technological innovation and urban-rural income gap.

Table 7. VECM Granger causality test

| Dependent variable | Independent variable  Short-run | |  |  | Long-run  [p-value] |
| --- | --- | --- | --- | --- | --- |
|  | F-statistic  [p-value] |  |  |  |  |
|  | △lnGI | △lnTI | △lnFIR |  | ECTt-1 |
| △lnGI | ­- | 13.824***  [0.0008] | 9.4808**  [0.0044] |  | -0.0003**  [0.035] |
|  |  |  |  |  |  |
| △lnTI | 7.4338**  [0.0107] | - | 7.5969***  [0.0098] |  | -0.0015**  [0.006] |
|  |  |  |  |  |  |
| △lnFIR | 5.0151**  [0.0327] | 4.2866**  [0.0471] | - |  | -0.0010***  [0.000] |

Note: *** and **denote significance at 1% and 5% levels, respectively.

Overall, short-term fluctuations and long-term equilibrium characterize the relationship between financial development, technological innovation, and the urban-rural income gap. Financial development and technological innovation will have an impact on the urban-rural income gap, which is the result of China's financial development bias, and it is also an inevitable phenomenon that the process of technological innovation and development has an impact on the income distribution pattern of different groups.

The bias in financial development has had an impact on the expansion of the urban-rural income gap in China to a certain extent. On the one hand, the profit orientation of capital and China's financial policies focus on supporting urbanization, resulting in a part of rural funds entering the urban financial market, accelerating the economic development of cities and the increase of urban residents' income, but not affecting rural construction, hindering the development and growth of the rural economy. On the other hand, in the case of rural finance lagging behind urban financial development, due to the imperfect rural financial market and mechanism, the level of rural investment and consumption is low, which is not conducive to the sustainable development of the cause of agriculture, rural areas and farmers and reduces the income of rural residents. Coupled with the government's rural financial support for agricultural development being relatively weak, the serious degradation of financial institutions to support agriculture, financial institutions in rural areas 'retreating weaker and weaker, weaker and retreating' phenomenon, leading to the rural financial market into a small scale, low-efficiency development dilemma. These long-term constraints have made it very difficult to narrow the income gap between urban and rural residents and gradually separated from poverty alleviation and rural revitalization.

The different effects of financial development on state-owned enterprises and private enterprises are also closely related to the urban-rural income gap. Since the reform and opening up, China has adopted 'gradual reform', and one of the important strategies is the dual track reform strategy, that is, plan and market, state-owned enterprises and private enterprises coexist. In the early days of reform and opening up, the government took supporting the development of state-owned enterprises as its primary goal, and also actively provided policy support for the development of small and medium-sized enterprises and private enterprises. However, with the deepening of the reform, the problem of low operating efficiency of state-owned enterprises has gradually emerged. To support the continued operation of state-owned enterprises, government departments have provided disguised subsidies to state-owned enterprises in various ways. The bias of financial policies toward state-owned enterprises will inevitably lead to more difficult living spaces for small and micro enterprises or private enterprises. This shows that the services currently provided by China's financial industry cannot fully meet the financing needs of private enterprises and small and medium-sized enterprises, and the financial system still has obvious shortcomings. Although the dual-track reform is intended to support the common development of state-owned enterprises and private enterprises, the actual implementation of the financial policy still prefers state-owned enterprises, and SMEs can not be treated equally. Small and micro enterprises and private enterprises are difficult to promote their development by financial means, which is not conducive to alleviating the income gap.

Due to the 'urban-rural dual' structure of the national industrial policy, there are differences in scientific and technological innovation ability and innovation efficiency between urban and rural areas.

Agricultural scientific and technological innovation does not match human capital, the accumulation rate of agricultural high-quality human capital lags behind the needs of technological innovation, and agricultural technological innovation has a weak impact on the urban-rural income gap; The urban industrial sector and science and technology service sector have an enormous investment in innovation resources, high scientific and technological innovation ability, high innovation efficiency, large profit space, and rapid production efficiency improvement. In addition, the dual economic structure of urban and rural causes the endowment of household resources and the education level of farmers less than in the city, which affects the increase of rural residents' income and increases the income gap between urban and rural residents.

Thanks!

**Reviewer #1comments5:**

The study should be summarized in just THREE sentences at the beginning of the conclusion.

**Response:** Thank you very much for your reminder. We have summarized the conclusion in three sentences at the beginning of this manuscript. Please see the revised version of manuscript for details.

Thanks once more!

**Reviewer #1comments6:**

The policy recommendations are vague. One does not need to carry out an entire study to arrive at these policy recommendations.

**Response:** Thanks for your valuable comments and suggestions. We have deleted, supplemented, and revised the corresponding policy recommendations based on the research conclusion of this manuscript. The details are as follows:

**Conclusion and policy suggestion**

There is no relevant research on the relationship between financial development, technological innovation and urban-rural income gap in the existing literature. In the current environment of steady economic development and building a harmonious society, narrowing the urban-rural income gap is an important problem in the process of economic development in China. Therefore, this study investigates the FKC for urban-rural income gap in case of China for the period of 1985-2019. This study has intensified on the technological innovation-income gap link with the FKC. For this purpose, we apply the ARDL approach and Johansen method for cointegration. In addition, the long run coefficient estimates are conducted by DOLS, FMOLS and CCR estimators. We also apply the VECM Granger procedure to causality. Finally, we using OLS regression analysis to variables. Compared with existing research, this study has made improvements in the following two aspects: This paper will add the indicator of technological innovation. On the one hand, there is no literature on the relationship between the three in China. On the other hand, technological innovation is closely related to financial development and the income gap between urban and rural residents. Thus, the level of technological innovation cannot be abandoned in the study; It will verify the FKC theory and analyze the link between technological innovation and urban-rural income gap under the FKC hypothesis.

It is found that the long-run relationship exists among the variables under the structural breaks. The main finding obtained from the long-run coefficient estimates reveal that technological innovation increases urban-rural income gap. The findings confirm the validity of the FKC hypothesis for China’s economy in the long run. The causality analysis shows a bi-directional causality between financial development, technological innovation and urban-rural income gap in the long run. We can present the following policy suggestions for China’s economy.

First, expand the scale of rural financial development and improve the efficiency of rural financial development. Because of the difficulty of loans in rural areas and the outflow of rural funds from rural areas through household savings and savings of township enterprises, when farmers have financing needs, they cannot get corresponding financing support because they cannot meet the credit threshold of financial institutions, and the efficiency of rural financial allocation is low. Therefore, the government should establish a sound agricultural financial system, optimize the share of agricultural financial services and financial resources, and try to improve the uneven distribution of financial resources between urban and rural areas and financial development to benefit more high-income people and less rural residents. Increase preferential policy support for rural economic development, improve the rural financial organization system, and promote agricultural modernization. In addition, the continuous expansion and upgrading of rural agricultural financial services will help attract more financial institutions and financial products to enter the countryside, and will also help rural areas reduce financing costs and thresholds, and reduce the loss of rural financial resources and talents. The improvement of rural financial operation efficiency can convert the absorbed rural savings into rural loans in real time, increase support for 'agriculture, rural areas and farmers' funds, increase the utilization rate of rural financial resources, and improve the development of rural financial development.

Second, Financial regulators should appropriately relax the market access threshold, encourage rural commercial banks, small and medium-sized commercial banks, financing guarantee companies, and other institutions to take root in the countryside, form a diversified rural financial service system that is competitive and coexisting with each other, and constantly improve the financial availability of farmers.

Third, adjust the structure of financial development, improve the efficiency of financial development, and narrow the income gap between urban and rural residents. We should constantly improve the financial market, establish a perfect and multi-level financial system that adapts to the development of the times, and set up corresponding regulatory authorities to give full play to the independent effect of the financial market. In terms of the income distribution, we should not only focus on efficiency but also fairness in distribution. So should financial development. We should pay attention to the efficiency and structure of financial development while expanding the scale of financial development. The focus of financial development before was mainly on expanding the scale, and always ignored the efficiency of financial development. Therefore, while developing finance, we should take into account the expansion of development scale and efficiency improvement, but we can not ignore the rationality of financial development structure.

Last, the government should take practical measures and introduce preferential policies to support technological innovation of small and medium-sized enterprises, create good financing conditions for SMEs, and establish and develop technological service systems for SMEs. In addition, local governments should insist on promoting industry to agriculture in technological innovation, cities supporting rural areas, and continuously increasing the intensity of financial investment in science and technology. On the one hand, while creating a good background to promote technological innovation, it should also introduce and implement relevant policies to promote technological innovation, create special innovation funds, actively subsidize and encourage scientific and technological innovation in large, medium, and small enterprises, research and development institutions, universities and other major scientific and technological innovation achievements. Vigorously promote industry-university-research cooperation between enterprises and universities, and help enterprises expand the market for technological innovation products and services. On the other hand, actively establish and improve the agricultural technology innovation coordination mechanism, improve the construction of agricultural technology innovation software and hardware, raise the level of agricultural technology, and lay a solid foundation for the construction of new rural industries.

This paper has some limitations. First, since we strengthen the relationship between technological innovation and urban-rural income gap in the context of FKC hypothesis, several explanatory variables such as globalization, human capital, and renewable energy are not included in our specifications. Second, many indicators of financial developments such as current liabilities and financial development index are not used for empirical analysis. At the same time, the study may inspire future researches. In this paper, there are some limitations in the relationship between patent licensing data analysis and urban-rural income gap. In the follow-up study, independent technological innovation indicators such as high-tech product export and R&D investment can be considered to further demonstrate the impact of technological innovation on urban-rural income gap and the differences in the results of different indicators. Therefore, future researches can investigate the impact of technological innovation on urban-rural income gap in detail and prefer comparative empirical results.

Thanks once more!

**Editors #comments1:**

Please review your reference list to ensure that it is complete and correct. If you have cited papers that have been retracted, please include the rationale for doing so in the manuscript text, or remove these references and replace them with relevant current references. Any changes to the reference list should be mentioned in the rebuttal letter that accompanies your revised manuscript. If you need to cite a retracted article, indicate the article’s retracted status in the References list and also include a citation and full reference for the retraction notice.

**Response:** Thank you very much for your reminder. We have rechecked and corrected the references in the manuscript. The details are as follows:

**References：**

1. Kuznets S. Economic growth and income inequality. The Economic Review.1995; 45(1),1–28
2. Greenwood J, Jovanovich B. Financial development, growth, and the distribution of income. Journal of Political Economy. 1990; 98(5), 1076–1107.
3. Zhenmei Hou, Maozai Tian, Zhihao Wang, Yan Dou. Study on the impact of technological innovation on the income gap between urban and rural residents—Based on the empirical analysis of Western Ethnic agglomeration. Practice and understanding of mathematics. 2020; 50(02): 53–64.
4. Pan X, Uddin M K, Ai B, Pan X, Saima U. Influential factors of carbon emissions intensity in OECD countries: evidence from symbolic regression. Journal of Cleaner Production. 2019; 220,1194–1201. <https://doi.org/10.1016/j.jclepro.2019.02.195>.
5. Huiping W, Meixia W. Effects of technological innovation on energy efficiency in China: evidence from dynamic panel of 284 cities. Science of The Total Environment. 2020; 709,136172. <https://doi.org/10.1016/j.scitotenv.2019.136172>.
6. Yii K-J, Geetha C. The nexus between technology innovation and CO_2_ emissions in Malaysia: evidence from granger causality test. Energy Procedia. 2017; 105,3118–3124. <https://doi.org/10.1016/j.egypro.2017.03.654>.
7. Townsend R M, Ueda K. Financial Deepening, Inequality, and Growth: A Model—Based Quantitative Evaluation. The Review of Economic Studies.2006; 73(1):251–293. <https://doi.org/10.1111/j.1467-937X.2006.00376.x>.
8. Clarke, George RG, Heng-fu Z, Lixin Colin X. Finance and income inequality: test of alternative theories. World Bank Publications. 2003.
9. Pradhan R P. The nexus between finance, growth and poverty in India: The cointegration and causality approach. Asian Social Science. 2010; 6(9):114.
10. Law S H, Tan H B, Azman-Saini W N W. Financial development and income inequality at different levels of institutional quality. Emerging Markets Finance and Trade. 2014; 50(sup1),21–33. <https://doi.org/10.1016/j.jclepro.2019.02.195>.
11. Sehrawat M, Giri, A K. Financial development and income inequality in India: an application of ARDL approach. International Journal of Social Economics.2015; 42(1):64–81. <https://doi.org/10.1108/IJSE-09-2013-0208>.
12. Haishu Qiao, Li Chen. Re test of the inverted U-shaped relationship between financial development and urban-rural income gap: An Empirical Analysis Based on China's County cross-sectional data. China's rural economy.2009; (07):68–76+85.
13. Zongyi Hu, Yiwen Liu. Research on Kuznets Effect of unbalanced financial development and urban-rural income Gap Empirical Analysis Based on cross-sectional data of counties in China. Statistical research.2010; 27(05):25–31.doi:10.3969/j.issn.1002-4565.2010.05.004.
14. Nan Yang, Chuoxin Ma. The dynamic inverted U evolution of the impact of China's financial development on the urban-rural income gap and the prediction of its decline point. Financial research. 2014; (11):175–190.
15. Zhiqiang Ye, Xiding Chen, Shunming Zhang. Can financial development reduce the income gap between urban and rural areas-Evidence from China. Financial research.2011; (02):42–56.
16. Fei Jia. Test on the unbalanced effect of financial development on urban rural income gap. Statistics and decision making. 2015; (24): 92–95.doi:10.13546/j.cnki.tjyjc.2015.24.026.
17. Yongqiang Sun, Yulin Wan. Financial Development, Opening Up and Income Gap between Urban and Rural Residents: An Empirical Analysis Based on Interprovincial Panel Data from 1978 to 2008. Financial Research. 2011; (01):28–39.
18. Youcai Yang. Financial Development and Economic Growth: An Analysis Based on the Threshold Variables of China's Financial Development. Journal of Financial Research. 2014; (02):59–71.
19. Edward E, Leamer. What’s the use of factor contents? Journal of International Economics.2000; 50(1). <https://doi.org/10.1016/S0022-19-96(99)00004-5>.
20. Antonio C. Agglomeration effects in European. European Economic Review.2002; 46:213-227. <https://doi.org/10.1016/S0014-2921(00)00099-4.>
21. Cirillo V, Corsi M, D’Ippoliti C. European households’incomes since the crisis. Investigaci6n Economica.2017; 76(301):57–85. <https://doi.org/10.1016/j.inveco.2017.12.002>
22. Nathan M, Lee N. Cultural Diversity, Innovation, and Entrepreneurship: Firm-level Evidence from London. Economic Geography.2013; 89(4):367–394.
23. Aghion P, Akcigit U, Bergeaud A, Blundell R, Hémous D. Innovation and top income inequality. The Review of Economic Studies.2019; 86(1),1–45. <https://doi.org/10.1093/restud/rdy027>.
24. Yong Chen, Zhe Bai. Skill-biased technological progress, labor agglomeration effect and widening of regional wage gap. China Industrial Economy. 2018; (09):79–97. doi:10.19581/j.cnki.ciejournal.2018.09.015.
25. Peng Zeng, Gongliang Wu, Xiaojun Zang. Research on the Relationship between Technological Progress, Urbanization and Urban-Rural Income Gap: Based on empirical analysis of Urban Agglomerations in China. East China Economic Management. 2016; 30(02):64–70.
26. Zhiqing, Dong, Xiao Cai, Linhui Wang. Skill Premium: an explanation based on the direction of technological progress. Chinese Social Sciences. 2014; (10):22–40+205-206.
27. Lei Ma. Human capital structure, technological progress and urban rural income gap: an analysis based on the panel data of 30 provinces in China from 2002 to 2013. East China economic management. 2016; 30(02):56–63.doi:[10.3969/j.issn.1007-5097.2016.02.010](http://dx.chinadoi.cn/10.3969/j.issn.1007-5097.2016.02.010).
28. Dickey D A, Fuller, W A. Likelihood ratio statistics for autoregressive time series with a unit root. Econometrica.1981; 49(4),1057–1072. https://doi.org/10.2307/1912517.
29. Phillips P C B, Perron P. Testing for a unit root in time series regression. Biometrika.1988; 75(2),335–346. <https://doi.org/10.1093/biomet/75.2.335>
30. Elliott G, Rothenberg, T J, Stock J H. Efficient tests for an autoregressive unit root. Econo-metrica.1996; 64(4),813–836. <https://doi.org/10.2307/2171846.>
31. Pesaran M H, Shin Y, Smith R. Bounds testing approaches to the analysis of level relationship. Journal of Applied Econometrics, 2001; (3):289-326. <https://doi.org/10.1002/jae.616>.
32. Stock J H, Watson M W. A simple estimator of cointegrating vectors in higher order integrated systems. Econometrica.1993; 61(4),783–820. <https://doi.org/10.2307/2951763>.
33. Phillips P C B, Hansen B E. Statistical inference in instrumental variables regression with I(1) processes. The Review of Economic Studies. 1990; 57(1),99–125. <https://doi.org/10.2307/2297545>.
34. Park J Y. Canonical cointegrating regressions. Econometrica.1992; 60(1),119–143. <http://www.jstor.org/stable/2951679.>
35. Engle R F, Granger C W J. Co-integration and error correction: representation, estimation, and testing. Econometrica.1987; 55(2), 251–276. <https://doi.org/10.2307/1913236>.
36. Jing Wang. An analysis of the relationship between Taiwan's economic openness and income gap—An Empirical Study Based on ardl-ecm model. Industrial economic circles. 2016; 03(02):164–172.
37. Bildirici M E. The effects of militarization on biofuel consumption and CO_2_ emission. Journal of Cleaner Production.2017; 152,420–428. <https://doi.org/10.1016/j.jclepro.2017.03.103>.
38. Fan Ye, Dongtao Zou, Xiheng Yuan. The impact of economic financialization on the income gap between urban and rural areas in China: an analysis based on provincial panel data from 1978 to 2013. Contemporary economic science. 2015; 37 (06):61–67+124.
39. Aghion P, Akcigit U, Bergeaud A, Blundell R, Hemous D. Innovation and top income inequality. Review of Economic Studies.2019; 86(1),1–45.<https://doi.org/10.1093/restud/rdy027>.
40. Mnif S. Bilateral relationship between technological changes and income inequality in developing countries. Atlantic Review of Economics.2016; 1(1),4.
41. Yingli Zhang, Zhengyong Yang. Mechanism and dynamic analysis of financial development and urbanization on urban and rural income gap. Statistics and Decision Making. 2018; 34(05):84-88. doi:10.13546/j.cnki.tjyjc.2018.05.020.
42. Antonelli C, Gehringer A. Technological change, rent and income inequalities: a Schumpeterian approach. Technological Forecasting and Social Change.2017; 115, 85–98. <https://doi.org/10.1016/j.techfore.2016.09.023>.
43. Zhihuan Chen, Hongge Zhu, Bo Cao. Financial Development, Economic Growth, and Energy Consumption Analysis is based on the ARDL-ECM model. Technical Economy and Management Research. 2020; (05):97–104.
44. Tao Feng, Maoguang Wu, Meisha Zhang. Financial development, industrial structure and urban-rural income gap: an analysis based on the perspective of "from real to virtual" finance. Exploration of economic problems. 2020; (10):170–181.
45. Shulan Fei, Jiqiang Guo. The impact of the statistical income attribution of migrant workers on the income gap between urban and rural areas. Statistical study. 2014; 31(06):17–24.
46. Jinpei Liu, Xiaoxia Song, Huayou Chen, Guanzhen Wang, Zhen Wang. Study on long-term equilibrium and causal dynamics of influencing factors affecting per capita carbon emissions in China by based on the empirical analysis of structural mutation ARDL-VECM model. Operations Preparation and Management. 2019; 28(09):57–65.
47. Ping Li, Tinghua Liu. Research on the Relations between Technology Innovation and Income Inquality—Based on China Data. Industrial Technical economy. 2009; 1:41–46.
48. Jingwen Li, Zhengdong Yang. The impact of technological innovation on industrial structure and income distribution. Journal of Social Science of Jilin University. 2013; (6):5–11.doi:10.15939/j.jujsse.2013.06.014.
49. Madhu S H W, Giri A K. Financial development and income inequality in India: an application of ARDL approach. Social Indicators Research.2011; 41(2):1-26. <https://doi.org/10.1108/IJSE-09-2013-0208>.
50. Huaizhong Mu, Peng Wu. Urbanization, industrial structure optimization and urban and rural income gap. Economist. 2016; (05):37–44.doi: 10.16158/j.cnki.51-1312/f.2016.05.006.
51. Pece A M, Simona O E O, Salisteanu F. Innovation and economic growth: an empirical analysis for CEE countries. Procedia Economics and Finance.2015; 26,461–467. <https://doi.org/10.1016/S2212-5671(15)008746>.
52. Permana M Y, Lantu D C, Suharto, Y. The effect of innovation and technological special-ization on income inequality. Problems and Perspectives in Management.2018; 16(4):51–63.
53. Risso W A, Carrera E J S. On the impact of innovation and inequality in economic growth. Economics of Innovation and New Technology. 2019; 28(1):64–81. <https://doi.org/10.1080/10438599.2018.1429534>.
54. Sohag K, Begum R A. Abdullah, S.M.S. et al. Dynamics of energy use, technological innovation, economic growth and trade openness in Malaysia. Energy.2015; 90 (Part2):1497–1507. <https://doi.org/10.1016/j.energy.2015.06.101>.
55. Shahbaz M, Raghutla C, Song M, Zameer, H, Jiao Z. Public-private partnerships investment in energy as new determinant of CO_2_ emissions: the role of technological innovations in China. Energy Economics.2020; 86,104664. <https://doi.org/10.1016/j.104664>.
56. Thornton J, Tommaso C D. The long-run relationship between finance and income inequal-ity: evidence from panel data. Finance Research Letters. 2020; 32,101180. <https://doi.org/10.1016/j.frl.2019.04.036>.
57. Yantao Zhang. Energy Consumption and Economic Growth Restudy—based on FMOLS, DOLS, CCR Research. Journal of North China University of Electric Power (Social Sciences). 2012; (01):5–9.
58. Cetin M, Demir H, Saygin S. Financial development, technological innovation and income inequality: Time series evidence from Turkey. Social Indicators Research.2021; 156(1),47–69. <https://doi.org/10.1007/s11205-021-02641-7>.
59. Ozturk S, Cetin M, Demir H. Income inequality and CO2 emissions: nonlinear evidence from Turkey. En-vironment, Development and Sustainability.2021; 1–18. <https://doi.org/10.1007/s10668-021-01922-y.>
60. Altunbaş Y, Thornton J. The impact of financial development on income inequality: A quantile regression approach. Economics Letters.2019; 175,51–56. <https://doi.org/10.1016/j.econlet.2018.12.030>.

Thanks!
